# Community Health Workers' Multiple Understandings of Schistosomiasis Transmission Pathways: Exploring Contextual Factors in Côte d'Ivoire, Kenya and Uganda

**DOI:** 10.1111/tmi.70195

**Published:** 2026-07-01

**Authors:** Dianne Verhoeven, Lisa Sophie Reigl, Alice Sereti Sinkeet, Mary Maghanga, Alexise Djouquou Gnahore, Bi Tra Jamal Sehi, Suzanne Lobohon Lavry, Andrea Buhl, Marie Frese, Jennifer Burrill, Eveline Hürlimann, Peter Steinmann, Nora Monnier, Ashley Preston, Janet Masaku, Matthias Richter, Mary Amuyunzu Nyamongo, Alain Toh, Stella Neema, Isabelle L. Lange, Andrea S. Winkler, the Pediatric Praziquantel Consortium

**Affiliations:** ^1^ Department of Neurology TUM University Hospital, and Center for Global Health, TUM School of Medicine and Health, Technical University of Munich Munich Germany; ^2^ African Institute for Health and Development Nairobi Kenya; ^3^ Department of Sociology Université Felix‐Houphouët Boigny Abidjan Côte d'Ivoire; ^4^ Institute of Social Anthropology, University of Basel Basel Switzerland; ^5^ Institute for Occupational and Maritime Medicine, University Medical Center Hamburg‐Eppendorf Hamburg Germany; ^6^ Unlimit Health London UK; ^7^ Swiss Tropical and Public Health Institute Allschwil Switzerland; ^8^ Eastern and Southern Africa Centre of International Parasite Control (ESACIPAC) Kenya Medical Research Institute (KEMRI) Nairobi Kenya; ^9^ Department of Health and Sport Sciences TUM School of Medicine and Health, Technical University of Munich Munich Germany; ^10^ Department of Sociology and Anthropology School of Social Sciences, Makerere University Kampala Uganda; ^11^ Department of Infectious Diseases Epidemiology London School of Hygiene and Tropical Medicine London UK; ^12^ Department of Community Medicine and Global Health Institute of Health and Society, University of Oslo Oslo Norway

**Keywords:** community health workers, contextual factors, Côte d'Ivoire, Kenya, schistosomiasis, transmission pathways, Uganda

## Abstract

**Objective:**

Schistosomiasis remains a public health concern in sub‐Saharan Africa, affecting over 250 million people. In endemic settings, schistosomiasis transmission is not always understood exclusively in biomedical terms. Community health workers (CHWs) contribute to schistosomiasis control through mass drug administration and health education, yet limited evidence exists on how they understand schistosomiasis transmission. This study explores how CHWs in Côte d'Ivoire, Kenya and Uganda explain schistosomiasis transmission and how these explanations relate to wider disease and training contexts.

**Methods:**

This paper draws on the CHW component of a larger mixed‐methods study conducted in Côte d'Ivoire, Kenya and Uganda, with multiple respondent groups. We present findings from interviews and focus group discussions with CHWs. Data were analysed thematically, at both semantic (descriptive) and latent (interpretive) level, employing Kleinman's framework of explanatory models and Good's work on semantic networks to support interpretation.

**Results:**

CHWs expressed multiple understandings of schistosomiasis transmission. Most described schistosomiasis transmission in biomedical terms, often emphasising skin contact with contaminated water. Others combined biomedical terms with locally circulating ideas about disease transmission, linking infection to drinking unsafe water, stepping on faeces, flies or other diseases such as trachoma or diarrhoeal illnesses. These understandings emerged in relation to a complex disease landscape in which signs, symptoms and perceived transmission routes overlapped. They were also situated within uneven training experiences, including irregular refresher sessions, variable access to schistosomiasis‐specific training and training focused mainly on treatment campaign logistics.

**Conclusion:**

CHWs' varying understandings of schistosomiasis reflect not only individual knowledge but also the broader disease landscape and training contexts in which they work. Strengthening schistosomiasis communication therefore requires attention to how CHWs interpret, adapt and combine disease‐specific information with other sources of knowledge, and to how training can better prepare them to navigate similarities and differences between disease‐specific messages in everyday practice.

## 1 Introduction

Neglected tropical diseases (NTDs) are diseases of poverty, thriving where sanitation, clean water, and access to health care are scarce [1]. These diseases disproportionately affect poor populations living in tropical and sub‐tropical regions [2]. Among NTDs, schistosomiasis—a waterborne parasitic infection also commonly known as bilharzia—is particularly prevalent, affecting over 250 million people, primarily in Sub‐Saharan Africa (SSA), including a significant number of children [3, 4].

Schistosomiasis occurs when schistosomes larvae released from freshwater snails penetrate human skin during contact with contaminated water, subsequently developing into adult schistosomes within the human body [5]. Freshwater bodies become infested when infected individuals urinate or defecate in these waters, releasing eggs that hatch miracidia that find hosts in snails [5], thereby continuing the cycle. Preventive chemotherapy in the form of mass drug administration (MDA) and health education are key strategies for controlling schistosomiasis [2, 5].

In endemic regions, schistosomiasis is not always interpreted exclusively in biomedical terms. Studies across SSA countries, including Tanzania, Kenya, Ghana, Côte d'Ivoire, Nigeria and Uganda, show that people living in endemic communities sometimes explain schistosomiasis transmission by drawing on a combination of biomedical information, bodily experiences, symptoms and socio‐cultural beliefs [6, 7, 8, 9]. In this article, we refer to these coexisting explanations as multiple understandings of schistosomiasis transmission: biomedical, experiential and socio‐cultural ways of explaining how the disease is acquired. These interpretations of schistosomiasis are often described in the literature as knowledge gaps or misconceptions. Although such framings are useful for identifying divergences from biomedical explanations, they do not fully explain how such interpretations are formed, why they persist, or how they are shaped by the social and environmental contexts in which people live [10, 11].

In contexts where multiple understandings of schistosomiasis coexist, community health workers (CHWs), who serve as frontline healthcare workers in underserved communities, play a central role in the provision of community‐based health services, including health education [12, 13, 14]. CHWs are recruited from the communities they live in and may work as volunteers receiving incentives, or as salaried staff, with training and supervision varying across health programmes and national contexts [14, 15, 16]. As key sources of health information, CHWs are involved not only in communicating biomedical messages, but also in mediating how these messages are understood within the communities they serve [17]. Their interpretations of schistosomiasis are therefore important because they may shape community understandings and preventive behaviours [18]. At the same time, because they are embedded within the communities they serve, CHWs may also draw on locally shared explanations of the disease. Yet, there is little evidence on how CHWs themselves explain schistosomiasis transmission or on the contexts through which these explanations are formed.

Examining how CHWs interpret schistosomiasis transmission is especially relevant in the context of intervention efforts that rely on them for community mobilisation, health education and treatment delivery. In this article, we examine how CHWs in Côte d'Ivoire, Kenya and Uganda explain schistosomiasis transmission, before situating these accounts within wider disease and training contexts that emerged in the data. In doing so we consider what these accounts reveal about the translation and adaptation of biomedical knowledge within intervention‐saturated community health settings. Rather than offering a direct country comparison, the cross‐country design is used to illustrate how multiple interpretations of schistosomiasis transmission emerge across endemic settings in both similar and locally specific ways. We show how global health messaging and everyday explanatory frameworks coexist, interact, and sometimes blur together, with implications for how prevention and treatment messages are communicated to communities.

## 2 Methods

### 2.1 Study Design

This study forms part of a broader formative research conducted within ADOPT, an implementation research programme of the Pediatric Praziquantel Consortium that aims to inform the introduction of a new paediatric treatment for schistosomiasis in preschool‐aged children (PSAC; children under 5 years of age) in endemic settings including Côte d'Ivoire, Kenya and Uganda [19]. ADOPT examines the social, programmatic and delivery contexts, including delivery platforms, that may shape the acceptability, feasibility and equitable reach of this new treatment. CHWs' explanations of schistosomiasis are relevant to their role as key actors in health education and may inform how interventions are communicated and implemented at the community level. The broader formative study was conducted before the pilot distribution of the new paediatric treatment and included parents, community leaders, district authorities, national‐level actors and CHWs. This paper focuses on qualitative data collected with CHWs, whereas being informed by contextual insights from the overall fieldwork with all participant groups.

### 2.2 Study Setting

The study was conducted in rural and peri‐urban locations in Côte d'Ivoire, Kenya and Uganda, which were selected for the pilot roll‐out of the new paediatric treatment. These settings were selected as part of the broader ADOPT implementation research programme and reflect areas where schistosomiasis remains endemic. In Côte d'Ivoire and Kenya, one location in each country had also served as a site for the phase‐2 and phase‐3 clinical trials of the new paediatric treatment conducted between 2016 and 2021 [20, 21]. The study areas have varying prevalences of the two primary types of schistosomiasis: intestinal and urogenital [4]. Livelihood and water‐use patterns are included to provide contextual background on everyday activities and forms of water contact that are relevant to local explanations of schistosomiasis transmission.

In Côte d'Ivoire, three study districts were selected: Agboville in the southeast, and Bangolo and Man in the west, the latter being the clinical trial site. Intestinal schistosomiasis is the most prevalent type in the western site [22, 23] while urogenital schistosomiasis is more common in the south [24]. Residents in these study sites primarily cultivate cocoa and coffee as cash crops alongside food crops and rely on dug wells, boreholes, taps, rivers and ponds for their water supply [22, 23, 25].

In Kenya, the study sites included Suba North Sub‐County in Homa Bay County a clinical trial site bordering Lake Victoria, where intestinal schistosomiasis is common [26]–and Matuga Sub‐County in Kwale County on the coast, where urogenital schistosomiasis prevalence is high [27]. Suba North residents depend mainly on Lake Victoria for water and fishing for income [26], whereas Matuga residents use dams and seasonal streams for water and engage in subsistence farming, cultivating crops including maize and cassava, and cash crops such as coconuts and mangoes [28].

In Uganda, two study sites were selected: Bugiri District in eastern Uganda, which borders Lake Victoria, and Hoima in western Uganda along the shoreline of Lake Albert. In both sites, intestinal schistosomiasis is more prevalent [29]. Fishing is the main livelihood in Hoima's lakeshore communities, whereas in Bugiri residents engage in food crop production, rice farming and fishing. In both locations, lake water is used for hygiene and laundry, and borehole water mainly for drinking and cooking.

### 2.3 Conceptual Framework

To interpret the patterns identified in CHWs' explanations of schistosomiasis, we drew on Arthur Kleinman's concept of explanatory models and Byron Good's notion of semantic networks. Explanatory models refer to how individuals make sense of illness, including its causes, modes of transmission, symptoms and treatment. These models are shaped by everyday experiences, social interactions and broader cultural and institutional contexts, and may differ from biomedical explanations [11]. Complementing this, the concept of semantic networks highlights how meanings of illness are not isolated but connected through language, bodily experiences, emotions and social relations [30]. Together, these frameworks informed our interpretation of how CHWs' understandings of schistosomiasis transmission are formed and how different forms of health knowledge coexist and interact.

### 2.4 Sampling, Recruitment and Data Collection Methods

We employed purposive sampling, a method that allows participants to be selected based on specific characteristics relevant to the research [31]. CHWs were purposively selected from settings participating in ADOPT, where the new paediatric treatment for schistosomiasis was expected to be introduced. As a participant group, CHWs were included in the wider formative study because their community‐based health roles and close relationships with community members positioned them to provide insight into local understandings of schistosomiasis and other implementation‐relevant issues [32]. Data collection methods, sample sizes, and combination of methods varied across countries in line with country‐level study design decisions and local implementation considerations. Within this design, focus group discussions (FGDs) provided group‐based data on how CHWs discussed schistosomiasis in Côte d'Ivoire, Kenya and Uganda, whereas in‐depth interviews (IDIs) in Uganda provided more individualised accounts.

Research activities were implemented by the University of Félix Houphouët‐Boigny in Côte d'Ivoire, the African Institute of Health and Development in Kenya, and Makerere University in Uganda, in collaboration with TUM University Hospital, which coordinated activities across the three countries. In Uganda, four research assistants and a supervisor conducted interviews, with authors LSR and MF contributing. In Kenya, eight research assistants and two supervisors per county were recruited in collaboration with county health teams. In Côte d'Ivoire, a six‐member team carried out interviews, with authors ADG and SLL also interviewing.

Table 1 summarises the data collection activities by country, methods, number of participants, date of data collection and the languages of interviews. In all three countries, a moderator and a note‐taker conducted the FGDs.

**TABLE 1:** Summary focus group discussions and in‐depth interviews conducted with community health workers in Côte d'Ivoire, Kenya and Uganda.

Country	Data collection method per district	Number of CHWs participating	Date of data collection	Languages used in interviews
Côte d'Ivoire	2 FGDs in Agboville	10	Dec 2021‐Jan 2022	French
	2 FGDs in Bangolo	11		
	2 FGDs in Man	11		
Kenya	2 FGDs in Homa Bay	23 (11 and 12)	April 2022	Swahili, Luo
	3 FGDs in Kwale	33 (12; 12; 9)		
Uganda	1 FGD in Bugiri	8	June 2022	English, Alur, Runyoro, Lusoga
	1 FGD in Hoima	6		
	8 IDIs	8		

The research teams in each country underwent a 2‐day training on the ADOPT project, data collection methods and tools, interviewing skills and ethical considerations regarding consenting and confidentiality safeguards. In total, 32 CHWs participated in the FGDs in Côte d'Ivoire, whereas 56 CHWs took part in the FGDs in Kenya. In Uganda, 8 CHWs participated in IDIs, and 14 in the two FGDs, resulting in a total of 22 participants.

### 2.5 Data Analysis

Interviews were audio‐recorded, and those originally conducted in local languages in Uganda and Kenya were translated into English, and all were transcribed. All transcripts underwent quality control, including checking translations where necessary, by the research assistants, field supervisors and the country's lead investigators.

Initial country‐level analyses used coding frameworks developed for all respondent types, with Uganda and Côte d'Ivoire data analysed in MAXQDA and Kenya manually. For this paper, we conducted a focused re‐analysis of CHWs' understanding of schistosomiasis transmission, informed by the synthesis of the country reports, and applying deductive codes on transcripts based on the research question and inductive codes to capture nuanced patterns emerging from the transcripts.

Data were analysed using thematic analysis, following semantic (descriptive) and latent (interpretive) themes [33]. In the first stage, we developed descriptive codes that captured CHWs' explanations of schistosomiasis transmission such as infection through water contact, ingestion of unsafe food or person‐to‐person transmission. In the second stage, we conducted an interpretive thematic analysis, examining broader patterns of meaning within and across countries. Kleinman's concept of explanatory models and Good's notion of semantic networks were not used to develop the coding framework, but were applied during interpretation to situate the findings within wider understandings of disease and illness [11, 30].

### 2.6 Ethical Considerations

Ethical approval for the study was obtained in all three countries. In Uganda the protocol was approved by Makerere University School of Social Sciences Research Ethics Committee (MAKSS‐REC 01.22.528) and the Uganda National Council for Science and Technology (no. SS1269ES). In Kenya, approval was provided by the Kenya Medical Research Institute's Scientific Ethics Review Unit and the National Commission for Science Technology and Innovation. In Côte d'Ivoire the protocol was approved by the Comité National d'Éthique des Sciences de la Vie et de la Santé (CNESVS) (193–21/MSHPCMU/CNESVS). Administrative clearance was also obtained in each country. The study protocol for the three countries received ethical approval from the Technical University of Munich's ethics committee (73/21 S). Written informed consent was obtained from each participant, all of whom were literate, after reading the information sheet and receiving verbal explanation of the study's purpose.

## 3 Results

The results first present the range of explanations CHWs gave for schistosomiasis transmission, followed by contextual findings on CHWs' accounts of schistosomiasis transmission in relation to other diseases and their training experiences. Country and site references are used to indicate where particular explanations appeared and to highlight relevant similarities and differences in how schistosomiasis transmission was articulated across settings.

### 3.1 Multiple Understandings of Schistosomiasis Transmission Among CHWs

The biomedical explanation for the transmission of schistosomes is that the parasite infects individuals when it penetrates the skin during contact with infested water. Across Côte d'Ivoire, Kenya and Uganda, most CHWs described schistosomiasis transmission as occurring through contact with contaminated water, particularly through walking, bathing, playing or swimming.

However, these biomedical explanations often coexisted with other accounts of how schistosomiasis enters or moves through the body. Across the study sites, these accounts can be grouped into five recurring themes: (1) ingesting unsafe water and uncooked or unwashed food; (2) penetration of schistosomes through wounds and other body openings; (3) direct contact with faeces and urine; (4) flies as carriers; (5) person‐to‐person transmission.

#### 3.1.1 Ingesting Unsafe Water and Uncooked or Unwashed Food

Several CHWs described schistosomiasis in relation to ingestion, linking infection to drinking untreated water or consuming unwashed or uncooked food. This explanation appeared in accounts from Uganda, Kenya and Côte d'Ivoire, although it was articulated in different ways across sites.

In some accounts, ingestion was combined with water‐contact explanations rather than presented as a completely separate route. In Hoima, Uganda, one CHW described schistosomiasis as entering the body both through stepping in dirty lake water and through drinking unboiled water, linking this to the stomach:

Bilharzia, like when we were still in school, they used to tell us that if you step in dirty lake water, the disease enters your body and that you can also get the disease when you drink unboiled water. That is how the disease enters our stomach. (IDI 1 with CHW in Hoima, Uganda)

A similar combination appeared in Homa Bay, Kenya, where one CHW referred schistosomiasis as *kichocho/ala remo* [urinating blood] and listed walking on wet ground or in water, bathing, drinking dirty water and drinking untreated water as possible routes of infection. This account named schistosomiasis through a visible symptom while also combining water‐contact and ingestion‐based explanations. However, the accounts differed in how far they elaborated the bodily process of infection. In Uganda, some CHWs framed schistosomiasis more specifically as a disease affecting the stomach. In an FGD in Bugiri, one CHW explained that because schistosomiasis was understood as ‘the disease of the stomach’, it could be acquired through drinking unboiled lake water.

Within the same FGD, another CHW connected this stomach‐related explanation to *kidaada*, a local term referring to stomach swelling that may be associated with schistosomiasis as well as with witchcraft through food. This CHW linked *kidaada* to the accumulation of germs from uncooked food:

Another thing which causes kidaada, I have always known, that if you are like, say, a child who eats uncooked food like potatoes, they have germs, and if these germs accumulate in the stomach, the stomach begins to swell. So those eating those uncooked food causes that disease. (FGD with CHWs in Bugiri, Uganda)

References to drinking unsafe water also appeared in Côte d'Ivoire, but these were brief and were not developed into a wider stomach‐ or food‐related explanation. Overall, ingestion‐based explanations ranged from general references to unsafe water or food to more locally elaborated interpretations linked to the stomach, stomach swelling and expressed through local terminology.

#### 3.1.2 Penetration of Schistosomes Through Wounds and Other Body Openings

A smaller number of CHWs described schistosomiasis as entering the body through wounds or other openings. This explanation appeared in accounts from Bugiri, Uganda and Kwale, Kenya.

In Bugiri, one CHW described this as information received from different health organisations:

[…] They [different organisations] told us that bilharzia, when you step in the water, there are germs which are like snails that enter you through wounds and they become a disease inside you. (IDI 1 with CHW in Bugiri, Uganda)

In the FGD in Bugiri, this explanation was extended beyond wounds to other body openings. One CHW suggested that schistosomes can enter the body through any part that allows things to enter or exit, such as the anus, ears, mouth and eyes. Another CHW linked women's exposure to schistosomiasis infection with water contact involving their genitals.

A related explanation also appeared in Kwale, Kenya, where CHWs discussed women's susceptibility to schistosomiasis during fishing or contact with dirty water. One CHW suggested that women could be affected because water could enter through the genital area:

I also second that women get affected by bilharzia because their private parts are open, so water just enters. (FGD 1 with CHW in Kwale, Kenya)

#### 3.1.3 Direct Contact With Faeces and Urine

A small number of CHWs across the three countries associated schistosomiasis transmission with direct contact with faeces or urine. These explanations linked infection to places where people urinate or defecate.

In Kenya, CHWs explained that both children and adults can become infected with schistosomiasis by walking barefoot in areas contaminated with urine, such as toilets:

According to our community, firstly, people need to understand what bilharzia is. It's a disease that spreads. A child in school can enter a place where the others urinate, and he can go there barefoot. That's one way to get contacted by the disease […]. (FGD 1 with CHWs in Kwale, Kenya)

In Uganda, a similar concern appeared in relation to open defecation. One CHW explained that when educating community members about schistosomiasis preventive measures, he advises people to avoid open defecation to reduce the risk of others stepping in faeces contaminated with schistosomes, which could penetrate the skin.

In Côte d'Ivoire, one CHW from Agboville described learning about schistosomiasis from a friend's experience with the disease. According to their account, the friend contracted schistosomiasis twice on the same day: once after he stepped in human faeces on his way to play soccer, and again later while swimming.

#### 3.1.4 Flies as Carriers of Schistosomiasis

A minority of CHWs in Bugiri, Uganda, viewed flies as the main carrier of schistosomiasis. These accounts linked transmission to open defecation, with flies as moving germs from faeces either to food, which could then be eaten, or elsewhere:

[…] When a person open defecates, especially those without pit‐latrines, it can easily cause bilharzia. A fly can get in contact with the stool and carry the virus sometimes a person with bilharzia is the one who has openly defecated so the fly can land on someone's food, and we just eat […]. (IDI 2 with CHW in Bugiri, Uganda)

In the same interview, the CHW also described snails as moving over faeces and leaving germs behind, which people could contract by stepping on contaminated ground. The fly‐carrier explanation therefore appeared alongside a broader account linking schistosomiasis to faecal contamination and direct contact with contaminated ground. In the FGD in Bugiri, two CHWs expressed a similar fly‐carrier view. One explained that schistosomiasis was not airborne but was instead carried in faeces and moved by flies and added he would welcome learning about ‘any other new way’ of transmission.

#### 3.1.5 Person‐To‐Person Transmission

A minority of CHWs in Kwale, Kenya described schistosomiasis as transmissible between individuals. These accounts included mother‐to‐child transmission and implied transmission between spouses or sexual partners.

One CHW suggested that schistosomiasis could be passed from an infected pregnant woman to her child, with signs of infection later appearing around the child's genital area. Another account implied transmission between sexual partners, describing a case in which a husband and both wives were found to have schistosomiasis.

Also, for us women, if your husband has bilharzia they can give it to you. I have referred a mother who had bilharzia and upon reaching the hospital, they called the husband, and the husband had a second wife. It was found that all of them had it. (FGD 1 with CHWs in Kwale, Kenya)

Across these themes, CHWs' accounts show that explanations of schistosomiasis transmission were not always mutually exclusive. Biomedical descriptions of water contact often appeared alongside other explanations involving ingestion, bodily openings, areas contaminated by faeces or urine, flies as carriers or interpersonal transmission. These accounts varied in detail across sites but together illustrate the range of coexisting understandings of transmission present in the study data.

### 3.2 Contextualising CHWs' Accounts of Schistosomiasis Transmission: Other Diseases and Training Experiences

Having described the multiple ways in which CHWs explained schistosomiasis transmission, this subsection turns to wider disease and training contexts in which schistosomiasis knowledge was described. It considers how CHWs discussed schistosomiasis in relation to other familiar diseases, local illness terms and uneven or indirect exposure to schistosomiasis‐related training. These contextual findings complement the transmission‐focused analysis above by showing how CHWs also described schistosomiasis knowledge in relation to other diseases and training experiences.

#### 3.2.1 Schistosomiasis in Relation to Other Familiar Diseases

In several sites, CHWs described schistosomiasis in relation to other diseases that were familiar in their communities. These accounts did not always indicate simple confusion. Rather, they showed how schistosomiasis could be discussed alongside other local disease categories, symptoms and transmission routes.

In Bugiri, Uganda, one CHW described a situation in which they recognised signs they associated with *kidaada/*schistosomiasis, but the child's mother described the condition as *lwanyaja* (diseases from the lake). The CHW explained that the presence of many lake‐related diseases made it difficult to distinguish between illness categories:

In fact, madam, since we are neighbouring the lake, we have many diseases. We have a disease called lwanyaja (of the lake); we have normal diarrhoea […] My understanding becomes so low […]. I can check a child, and all the signs indicate it's kidaada, but the mother of the child is saying that it's lwanyaja, etc. They always mix them up. So that's why I wanted to hear from you; how do I distinguish whether this is kidaada […]? (FGD with CHWs in Bugiri, Uganda)

In Côte d'Ivoire, CHWs also related schistosomiasis to other diseases when describing their training experiences. In Agboville, CHWs explained that schistosomiasis had been discussed during a training session on onchocerciasis. One CHW recalled being told that the disease came from ‘a fly’ in the water, whereas also mentioning signs such as blood in urine and blood in stool:

During the campaign, […] we were told to tell the parents that this disease comes from a fly, as she just mentioned, a fly that is in the water. And when we urinate in the water, we do everything that is bad […] the child might urinate blood, might have blood in the stool […]. This fly can cause other diseases in the child. (FGD 2 with CHWs in Agboville, Côte d'Ivoire)

In Bangolo, three CHWs similarly described schistosomiasis using signs/symptoms and transmission routes associated with other fly‐transmitted diseases. One CHW stated that schistosomiasis ‘affects the eyes a lot’, a symptom not associated with schistosomiasis. Another linked transmission of schistosomiasis to ‘the bite flies’, whereas a third specified tsetse flies, which are vectors of trypanosomes.

Together, these accounts show that CHWs sometimes discussed schistosomiasis in relation to other familiar diseases, local illness terms, symptoms and training messages.

#### 3.2.2 Uneven and Fragmented Training Experiences on Schistosomiasis

CHWs' accounts also pointed to varied exposure to schistosomiasis‐related training. In Uganda, some CHWs described MDA‐related responsibilities that did not necessarily involve formal training. For example, two CHWs from Bugiri, whose role was to announce upcoming MDA campaigns to treat schistosomiasis, reported not receiving training. Instead, they were informed about the disease through word of mouth from community drug distributors who had received training:

I always see the people who give it [the medication/praziquantel] out, they first go for seminar, and they train them how to do it. […] I don't participate in that, I have never. (IDI 3 with CHW in Bugiri, Uganda)

Where schistosomiasis‐related training was reported, CHWs often described it as brief and focused on the logistics of drug administration. In Bugiri, Uganda, CHWs who received a 1‐day training on schistosomiasis described it as primarily focused on the logistics of administering praziquantel such as measuring height to determine dosage. Some CHWs expressed a desire for a longer, more comprehensive program, in one case suggesting up to a month, including topics like identifying snail hosts and responding to community questions. However, one CHW felt additional training was unnecessary because the task of administering the drug remained the same.

In Hoima, Uganda, CHWs similarly described training as focused mainly on implementation logistics rather than broader disease knowledge or risk communication. One CHW, who coordinates other CHWs in his village in Hoima, shared his experience regarding the irregularity of follow‐up training and limited educational materials:

[…] Sometimes they train us and do not give us feedback. For example, they train you on bilharzia and they never come back. We need refresher training […] You cannot train me on something and take years to come back [for another training]. And, they train us and do not give us materials to use. (IDI 2 with CHW in Hoima, Uganda)

The same CHW also criticised the compressed training schedule which he felt disadvantaged CHWs with less formal education and limited their ability to understand and apply what was taught.

In Côte d'Ivoire, schistosomiasis was sometimes discussed within training on other diseases rather than through dedicated training. During an FGD in Bangolo, CHWs reported that schistosomiasis had been addressed briefly during a broader training on trachoma.

These accounts show that CHWs described schistosomiasis knowledge in relation to other familiar, overlapping symptoms and transmission routes, and varied training experiences related to schistosomiasis.

## 4 Discussion

This study explores how CHWs in Côte d'Ivoire, Kenya and Uganda explained schistosomiasis transmission, and how these explanations reflected multiple sources and forms of health knowledge. This is important given CHWs' central role in community health education. The findings show that CHWs held multiple coexisting understandings of how schistosomiasis is transmitted. In this discussion, we first interpret how CHWs made sense of schistosomiasis transmission through Kleinman's concept of explanatory models and Good's notion of semantic networks, before considering the contextual factors that may help explain why multiple and sometimes inconsistent explanations emerged.

### 4.1 Assembling Knowledge: How Do CHWs Make Sense of Schistosomiasis Transmission?

CHWs in our study articulated a range of explanations of how schistosomiasis enters the body. These diverse explanations were present both within and across Côte d'Ivoire, Kenya and Uganda, and sometimes coexisted in the accounts of individual CHWs, reflecting partially overlapping ways of understanding schistosomiasis transmission. Although some explanations aligned with biomedical descriptions of schistosomiasis transmission through skin contact with contaminated water, others reflected alternative pathways such as ingesting unsafe water or unwashed food, germs entering body openings, direct contact with faeces and urine, flies as carriers or person‐to‐person transmission. This range of explanations suggests that CHWs' understandings cannot be reduced to a single explanatory model of schistosomiasis transmission.

Similar explanations of schistosomiasis transmission have been reported in other endemic settings, including transmission through ingestion of unsafe water or food, direct contact with urine and faeces, flies as carriers and sexual transmission [6, 7, 9, 18, 26, 34, 35, 36]. Related analyses of the ADOPT formative study focusing on caregivers of PSAC in Kenya and Uganda also identified a variety of understandings of schistosomiasis transmission resembling those observed among CHWs in this study [37, 38]. Although these findings derive from distinct participant groups and cannot be directly compared, they suggest that the plurality observed among CHWs may align with broader patterns of how schistosomiasis is conceptualised at community level.

This alignment may reflect CHWs' dual position as community members and providers of health information [39]. As such, their accounts suggest that they did not simply reproduce community understandings of schistosomiasis, but assembled explanations from both locally shared ways of making sense of illness and biomedical explanations of schistosomiasis.

This process of assembling explanations is also evident in the language CHWs used, which reflected not only biomedical messages but also locally familiar ways of making sense of schistosomiasis. Terms such as ‘dirty’, ‘unboiled’, ‘uncooked’ and ‘untreated’ were used to describe water or food linking schistosomiasis to ingestion and to stomach‐related symptoms such as abdominal swelling. In Homa Bay, Kenya, the use of *kichocho/ala remo* [urinating blood] similarly shows how schistosomiasis could be named and understood through visible symptoms while also being explained through both water‐contact and ingestion‐based pathways. In Bugiri, Uganda, the local term *kidaada* was used to describe schistosomiasis but also refers to stomach swelling attributed to witchcraft through food poisoning. Although CHWs in Uganda did not explicitly describe schistosomiasis as caused by witchcraft, the use of the local term *kidaada* shows how biomedical explanations are shaped by existing cultural disease terms. These associations illustrate Good's concept of semantic networks, in which meanings of disease are formed through connections between symptoms, perceived causes, language and lived experience [30].

More broadly, the presence of multiple understandings of schistosomiasis transmission aligns with Kleinman's notion of explanatory models—ways of explaining illnesses that are shaped by socio‐cultural, political, ecological and historical contexts [11]. This perspective helps interpret CHWs' accounts not as isolated misconceptions, but as contextually situated explanations that may coexist and overlap as CHWs draw on different sources of knowledge.

What is noteworthy is not simply that some explanations diverged from the biomedical transmission model, but how CHWs assembled biomedical messages, local disease terms and everyday experience into multiple coexisting understandings of schistosomiasis transmission. Rather than reflecting confusion alone, this plurality points to an interpretive process through which CHWs make sense of schistosomiasis.

### 4.2 Situating CHWs' Explanations of Schistosomiasis Transmission: Disease Landscapes, Training and Programme Contexts

The coexistence of multiple understandings of schistosomiasis among CHWs can be understood in relation to the structural and contextual conditions in which they are trained and carry out their roles. In particular, the disease landscape, together with the organisation of training and programme delivery, may help explain how CHWs interpret health information and why multiple understandings of schistosomiasis transmission emerge.

First, the disease landscape in which CHWs work may make it difficult to maintain clear distinctions between schistosomiasis and other co‐endemic diseases. Schistosomiasis coexists with other prevalent NTDs targeted by preventive chemotherapy—such as soil‐transmitted helminths (STH), onchocerciasis and trachoma—as well as with diarrhoeal diseases, many of which share overlapping signs and symptoms or transmission routes [40].

This is reflected in how CHWs described schistosomiasis transmission in terms of ‘stepping on faeces’ suggesting a blending of STH and schistosomiasis messages—an overlap likely reinforced by integrated MDA campaigns for both diseases in Kenya and Uganda [41, 42]. Similarly, references to flies or open defecation reflected information more typical of trachoma or diarrhoeal disease education. In Côte d'Ivoire, some CHWs associated schistosomiasis with signs, symptoms or vectors more typically linked to onchocerciasis or trypanosomiasis. Such associations point to the difficulty of distinguishing between conditions that may be encountered through different disease programmes or recognised locally through similar bodily signs and symptoms. Although these messages encourage generally positive hygiene behaviours, they may also limit recognition of schistosomiasis‐specific risks such as contact with infested water [18, 43]. These examples suggest that CHWs' explanations are situated within a disease and programme context where several conditions share similar features and are addressed through multiple health activities.

Second, multiple coexisting understandings of schistosomiasis may become more likely in contexts where CHWs receive limited, irregular or logistics‐focused training with few opportunities to clarify disease‐specific transmission. In our study, for example, some CHWs in Uganda were excluded from trainings because their role was limited to mobilising for MDA, whereas those who attended reported focus on logistical procedures rather than on schistosomiasis itself. Training was also described as infrequent and lacking refresher opportunities, consistent with existing literature suggesting that irregular and campaign‐based training can contribute to gaps and inconsistencies in knowledge of diseases [16, 44, 45, 46, 47, 48].

Similar concerns are reflected in a related ADOPT formative analysis from Uganda, where district health authorities emphasised the need for more appropriate CHWs' training and described financial constraints that could affect programme delivery, including training [49]. These training constraints may also reflect broader programme structures. The constraints of MDA—such as funding gaps and the logistical demands of organising nationwide campaigns—may shape the scope and content of training, pushing programmes to prioritise short, logistics‐focused sessions [2, 50]. In addition, wider patterns of CHW financing—marked by underinvestment in primary health care and reliance on externally funded, disease‐specific programmes—may contribute to fragmented, campaign‐based training models that run parallel to routine government health services [51]. Together, these constraints may help explain why CHWs' opportunities to reinforce schistosomiasis‐specific knowledge remain uneven and infrequent.

Overall, our findings suggest that CHWs' multiple coexisting understandings arise where biomedical messages about schistosomiasis transmission are interpreted alongside local understandings of disease and knowledge from other health conditions. In our study, this helps explain how different sources of knowledge were combined and blurred together in practice. Figure 1 presents a conceptual synthesis of how the disease landscape and training context may shape this interpretive process.

**FIGURE 1:**
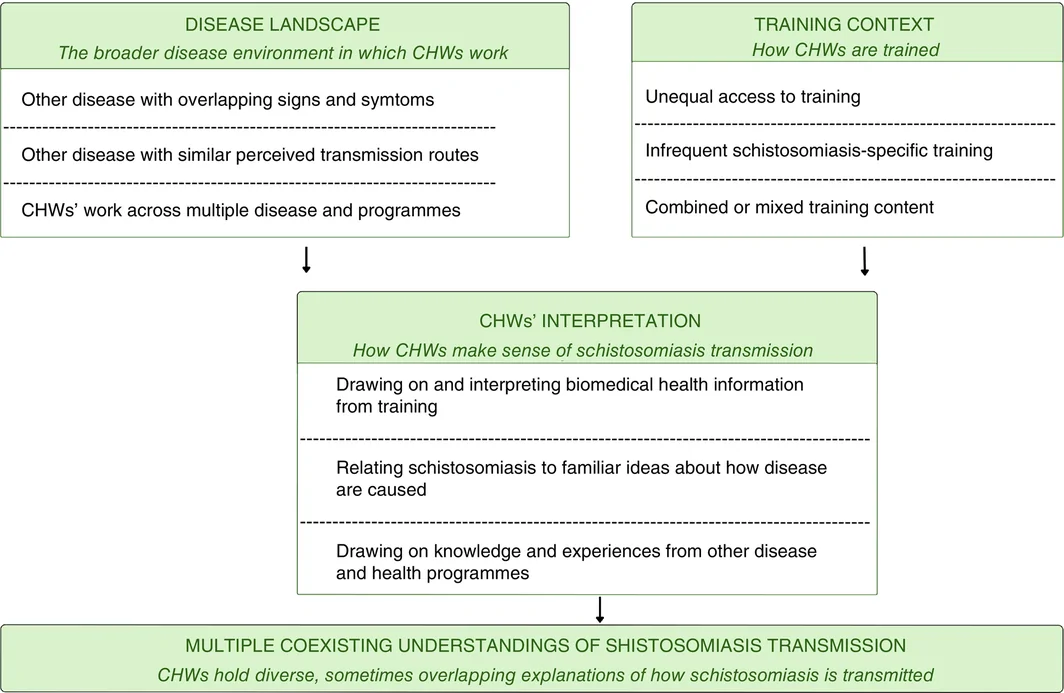
Conceptual synthesis of how CHWs' understandings of schistosomiasis transmission relate to the disease landscape and training context. The figure shows how CHWs' understandings are situated within the disease and training contexts in which they work. The disease landscape includes the presence of several diseases with similar signs, symptoms or perceived transmission routes, alongside CHWs' involvement in programmes addressing these conditions. The training context includes unequal or infrequent training, mixed training content and limited reinforcement of schistosomiasis‐specific messages. These contexts may contribute to situations in which biomedical explanations of schistosomiasis coexist with other familiar ideas about disease transmission.

### 4.3 Limitations

This study has several limitations. First, the analysis draws on qualitative data from a broader formative study. The depth of probing into CHWs' explanations of schistosomiasis transmission varied across sites and data collection methods, meaning that some explanations were explored in greater depth than others.

Second, data collection methods and sample sizes differed by country: FGDs were conducted in all three countries, whereas IDIs were conducted only in Uganda, and participant numbers varied by site. These differences may have influenced the type, volume and depth of accounts generated. In line with the analytic scope of the paper, we do not attempt direct country comparisons; instead, country and site references are used to indicate where particular explanations appeared and to interpret variation across settings cautiously.

Third, data were collected in multiple languages and translated for analysis. Although this process was supported by trained local research teams and collaborative analytic discussions, some nuance in local terminology, expressions or meanings may not have been fully captured in translation. To reduce this risk, key terms and interpretations were discussed and clarified with country teams during analysis.

Finally, the analysis is based on CHW data only. This allowed detailed examination of how CHWs explained schistosomiasis transmission, but data from other participant groups in the broader study were not formally analysed for this paper. As a result, the findings cannot fully address how CHWs' explanations relate to wider community understandings of schistosomiasis, or how other stakeholders understand CHWs' knowledge of transmission and their role in providing health information.

## 5 Conclusion

CHWs in Côte d'Ivoire, Kenya and Uganda held varied understandings of schistosomiasis transmission. Although most described transmission in ways that aligned with biomedical explanations, others expressed multiple or sometimes contradictory understandings. These variations should not be interpreted simply as individual knowledge gaps but as situated within the broader contexts in which CHWs work, including the disease landscape in which several diseases may share similar signs, symptoms or perceived transmission routes, and training contexts characterised by uneven access, limited schistosomiasis‐specific content and few refresher opportunities. Within these contexts, CHWs are not neutral messengers through whom biomedical knowledge flows unchanged. Rather, they may draw on different sources of knowledge, adapting and combining biomedical information with local illness terms, everyday experiences and knowledge from other diseases and health programmes.

Strengthening schistosomiasis communication therefore requires more than refining training content alone; it also requires attention to how CHWs interpret and apply health information in everyday practice. These findings also raise questions about what is realistically expected of CHWs' disease knowledge within intervention‐saturated settings. As shown in the results, training is often delivered through (vertical) programmes that prioritise disease‐specific messaging, yet CHWs operate within a more integrated everyday health landscape in which prevention advice is often blended, generalised and adapted. From this perspective, ‘inaccurate’ understandings of schistosomiasis transmission may not simply represent a deficit, but a pragmatic form of health reasoning shaped by fragmented training and the need to offer broadly protective guidance. This study therefore underscores the tensions between global health messaging and local contexts and points to the value of more holistic training approaches that equip CHWs to navigate a complex disease landscape, rather than narrowly reproducing programme‐specific messages.

## Funding

The Pediatric Praziquantel Consortium is financially supported by Merck, with in‐kind contributions from partners and grants from the Bill & Melinda Gates Foundation (2012), the Global Health Innovative Technology Fund (GHIT) (G2020‐102) (2014, 2015, 2016, 2019 and 2020), and the European & Developing Countries Clinical Trials Partnership (EDCTP) (RIA2019‐2895) as part of the EDCTP2 programme supported by the European Union (2018 and 2021). This manuscript, written on behalf of the Consortium, was made possible thanks to the support of these funders. The funders had no role in the study design, data collection and analysis, preparation of the manuscript or the decision to publish.

## Conflicts of Interest

The authors declare no conflicts of interest.

## Data Availability

Data from interviews cannot be made publicly available due to confidentiality, privacy, and ethical concerns. The qualitative data includes sensitive information that could identify participants or compromise their privacy. Moreover, the data was collected under an agreement of confidentiality with the participants, ensuring that their identities and the details shared would remain protected. We are committed to the principles of transparency and openness in research within the constraints of these ethical and legal obligations. Anonymized excerpts relevant to the study can be requested via neurologie@mri.tum.de.
